# Myocardium Metabolism in Physiological and Pathophysiological States: Implications of Epicardial Adipose Tissue and Potential Therapeutic Targets

**DOI:** 10.3390/ijms21072641

**Published:** 2020-04-10

**Authors:** Nerea Gandoy-Fieiras, Jose Ramon Gonzalez-Juanatey, Sonia Eiras

**Affiliations:** 1 Translational Cardiology Group, Health Research Institute, 15782 Santiago de Compostela, Spain; nereagandoy@gmail.com; 2 Cardiovascular Department, University Hospital of Santiago de Compostela, 15782 Santiago de Compostela, Spain; jose.ramon.gonzalez.juanatey@sergas.es; 3 Cardiology Group, Health Research Institute, 17582 Santiago de Compostela, Spain; 4 Centro de Investigación Biomédica en Red de Enfermedades Cardiovasculares (CIBERCV), 28029 Madrid, Spain

**Keywords:** myocardium, epicardial adipose tissue, metabolism, therapies

## Abstract

The main energy substrate of adult cardiomyocytes for their contractility are the fatty acids. Its metabolism generates high ATP levels at the expense of high oxygen consumption in the mitochondria. Under low oxygen supply, they can get energy from other substrates, mainly glucose, lactate, ketone bodies, etc., but the mitochondrial dysfunction, in pathological conditions, reduces the oxidative metabolism. In consequence, fatty acids are stored into epicardial fat and its accumulation provokes inflammation, insulin resistance, and oxidative stress, which enhance the myocardium dysfunction. Some therapies focused on improvement the fatty acids entry into mitochondria have failed to demonstrate benefits on cardiovascular disorders. Oppositely, those therapies with effects on epicardial fat volume and inflammation might improve the oxidative metabolism of myocardium and might reduce the cardiovascular disease progression. This review aims at explain (a) the energy substrate adaptation of myocardium in physiological conditions, (b) the reduction of oxidative metabolism in pathological conditions and consequences on epicardial fat accumulation and insulin resistance, and (c) the reduction of cardiovascular outcomes after regulation by some therapies.

## 1. The Metabolism of Cardiomyocytes in Physiological Condition

### 1.1. Excitation-Contraction of Cardiomyocytes

The transformation of rhythmic electrical stimulation into the production of mechanical force supports the excitation-contraction of cardiomyocytes. The electrical stimulation starts the trigger of depolarization that causes the opening of sarcolemma voltage-dependent calcium channels and, in consequence, a cytosolic Ca2+ entry. Other proteins involved in the action potential are the sodium and several types of potassium channels, the sodium–calcium exchanger, and the sarcolemma calcium ATPase [1,2]. Cardiomyocytes are constituted by myofibrils composed of sarcomeres. This is the minimum contractile unit, and it is constituted by thick and thin filaments with myosin or actin polymers, respectively. Ca2+ binds to troponin C in actin filaments. Afterwards, there is a conformational change and myosin heads bind to actin molecules. It constitutes a cross-bridge cycle. The myosin head uses energy in adenosine triphosphate (ATP) to move on the actin filament. In this way, it exerts the basic mechanical force for sarcomere shortening and contraction [3]. Each cross-bridge cycle needs one ATP. The decline of cytosolic Ca+2 due to the activity of adenosine triphosphatase (ATPase) in the sarcoplasmic reticulum, sarcolemma Na+/Ca2+ exchanger (NCX), and mitochondrial sequestration [2] produces the conclusion of the myofilament cross-bridge cycling and the beginning of sarcomere relaxation.

### 1.2. Energy and Substrates

ATP production in cardiomyocytes is generated from several substrates (carbohydrates, lipids, amino acids, and ketone bodies) into the mitochondria. The large amount of ATP required during the day justifies the important number of mitochondria per cell. Cardiomyocytes have an adaptive metabolic condition according to energy demand, substrate or oxygen supply, and regulation of mitochondrial biogenesis or fatty acids metabolism-involved genes. However, 60–70% of total energy is supplied by fatty acids oxidation [4], which consumes the highest oxygen concentration. The shift of fatty acid into glucose oxidation [5] improves the energy efficiency and contributes to metabolic benefits [6,7]. 

#### 1.2.1. Fatty Acids

The uptake of fatty acids into cardiomyocytes needs a carrier [8,9], named fatty acid-binding protein. Other proteins related to free fatty acids transport in cardiomyocytes are the fatty acid translocase (FAT/CD36) or fatty acid transporter protein 4 (FATP4) [10,11,12]. Although fatty acids constitute the main energy supply, cardiomyocytes have a low ability to synthesize and store them. Free fatty acids (FFA) are converted into fatty acyl-CoAs which can bind to acyl-CoA binding proteins. Afterwards, through carnitine palmitoyltransferase, FFA can go into the mitochondria and can be degraded in the β-oxidation pathway to generate acetyl-CoA by the tricarboxylic acid cycle. The resulting coenzymes, FADH2 and NADH, go to the electron transport chain with a consequent ATP generation and oxygen consumption.

#### 1.2.2. Glucose

Glucose, another substrate, can be degraded into lactate, stored into glycogen, or oxidized [13]. A 10–40% of the energy substrate comes from glucose-lactate oxidation. Glucose is transformed into pyruvate, which can be converted to lactate, decarboxylated to acetyl-CoA, or carboxylated to oxaloacetate or malate [14,15,16]. Although ATP production is higher from fatty acids oxidation, glucose oxidation is more efficient because it expends fewer molecules of oxygen. Thus, there is a shift from fatty acids to glucose substrate in acute high workload conditions [17]. The consumption of glucose will depend on fatty acids availability. Glucose is more important during the postprandial period because, at this time, an increase in insulin secretion upregulates glucose transporter, GLUT4, into membrane of cardiomyocytes. In the fetal heart, GLUT1 is the glucose transporters´ predominant isoform. This is important because the fetus developing needs glucose as energy supply. However, in the adult heart, cardiomyocytes prefer fatty acids to get energy, and GLUT4 isoform has a higher Michaelis constant (Km)—affinity—for transporting glucose in an insulin-dependent manner. 

Glucose is phosphorylated inside cells to prevent its exit and to initiate glycolysis. This metabolic pathway degrades the glucose-6-phosphate to get pyruvate, ATP, and NADH and H^+^. Glucose breakdown occurs in 10 steps that are grouped in the preparatory phase and payoff phase. Pyruvate is converted into acetyl-CoA that enters into the citric acid cycle. After each cycle, NADH, FADH2, and ATP are obtained. The resulting ATP will be used to collect Ca^2+^ to the sarcoplasmic reticulum during diastolic relaxation. Another pathway of the glucose-6-phosphate is the pentose phosphate pathway (PPP), which is responsible for the pentose phosphate production and the nucleotide and nucleic acid precursors and provides the reduction for biosynthesis of fatty acids or anaplerosis. This pathway is limited in the heart. There are pieces of evidence that this pathway can participate in pathophysiology [18]. Thus, high activation of this pathway, provoked by high glucose levels, inhibits the cardiomyocytes maturation. This process might explain some congenital heart disease in diabetic pregnancy [19]. Finally, glucose can be reduced into sorbitol by the polyol pathway which is controlled by aldose reductase (AR). Sorbitol through sorbitol dehydrogenase is oxidized into fructose to maintain the osmotic balance [20]. Myocardium does not store lipids or glycogen in large amounts. The alternative reserve is the phosphocreatine. The creatine joins the ATP to storage it. This energy can be used through creatine kinase activity. This enzyme is localized into sarcoplasmic reticulum and myofibrils for bringing ATP quickly. Mitochondrial creatine kinase is used to transport the mitochondrial ATP to cytosol. 

#### 1.2.3. Ketone Bodies

Other energy substrates are the ketone bodies which are formed in the liver and then are exported to organs. In normal conditions, acetone is formed in small amounts, but in starvation, untreated diabetes, or intense exercise conditions, its levels are higher. A high amount of acetone levels in blood might be toxic. The D-β-hydroxybutyrate is transformed into acetoacetate which reacts with an ester-CoA and reacts with succinyl-CoA, gaining access to the citric acid cycle.

### 1.3. Diet and Physical Activity

Diet and physical activity are the major factors involved in fatty acids or glucose oxidation. Fasting conditions increase released fatty acids by adipose tissue and their oxidation by cardiomyocytes. This process is associated with the inhibition of glucose oxidation [21]. However, after postprandial state, the high insulin and glucose increment [22] improve the intracellular glucose through insulin-dependent or independent glucose transporters (GLUT) [23,24] and there is an inhibition of fatty acids oxidation. Glucose can either be degraded into lactate, stored into glycogen, or oxidized [13] for getting energy. 

During physical activity, lactate can be used as an energy substrate [25] in muscle. The isozymes lactate dehydrogenase 4 (LDH4) and 5 (LDH5) transform pyruvate into lactate under high exercise situation or low oxygen levels. In the heart, the main isoforms are LDH1 and LDH2. They have more affinity for lactate than pyruvate, which will enter to the mitochondria [26]. The isometric exercise increases the blood pressure and concentric hypertrophy (wall thickness without chamber changes) of the heart. However, the isotonic exercise increments the production of catecholamines, and the regurgitation of aortovenous fistulae increase the contractility and eccentric hypertrophy. The physiological increment of heart size is an adaptation to provide more energy, oxygen, and blood to the body in high activity situation. There is an increase in cardiomyocytes size but not in number. Although these characteristics might remember a heart failure situation, some studies have suggested an increment of fatty acid and glucose oxidation [27], which is reduced in pathological conditions because of fibrosis and ventricle dysfunction [28]. Sex hormones and metabolic factors are implicated in exercise response, and the heart size is smaller in women than in men. This condition might implicate a differential metabolism condition associated with hormones regulation [29], but this point will be discussed below.

### 1.4. Circadian Rhythm

The center of circadian clock is in the hypothalamus, specifically in the suprachiasmatic nucleus. It is a 24-hour cycle which helps cells to anticipate the needs of the time of day and is regulated through feedback loops and zeitgebers as light or temperature. The gene expression oscillations in normal intact hearts can be modulated by extracellular (i.e., neurohumoral, workload, and circulating nutrients) and/or intracellular (i.e., circadian clock) stimulus. During the night, there is a reduction of vascular tone, oxygen demand, heart rate, and cell death [30]. The circadian cycle determines the expression of some genes. Organs and cells can have their own circadian core genes, named peripheral clock [31]. The circadian genes of cardiomyocytes are *BMA1*, *CLOCK*, *CRY*, and *PER* [32]. However, specific zeitgebers are still unknown [33]. During the day, blood pressure and heart rate variations are registered together with changes in glucose, fatty acids, and proteins metabolism [34]. Modifications in our daily routine, variations in sleep habits, low glucose level, or presence of norepinephrine dysregulate the circadian clock. However, adenosine monophosphate-activated protein kinase (AMPK), an energy-sensing kinase [35], and peroxisome proliferator-activated receptor gamma coactivator 1-alpha (PGC-1α), which is involved in mitochondrial biogenesis, can regulate the circadian genes [36]. Most of the metabolic enzymes are regulated by the circadian clock, like glucose-6-phosphate transport protein, pyruvate kinase, pyruvate dehydrogenase, glucose-6-phosphatase, acetyl-CoA carboxylase, cytochrome oxidase, lactate dehydrogenase, fatty acid synthase, etc. [37]. The cardiomyocyte circadian clock increases glucose uptake and its utilization through AMPK activation [38]. Glucose can also be stored into glycogen [39]. Other metabolites which are under the clock control are the ketone bodies [40]. During the early phase of sleep (rapid eye movement (REM)), there is a high sympathetic nervous system activity and, in consequence, blood pressure, body temperature, heart rate, and respiratory rate. However, during non-REM sleep, there is a lower metabolic rate. In consequence, the increase of circulating fatty acids improves the triglycerides storage. Just before waking in the morning, there is a peak of cortisol, the hormone produced by adrenal glands which is responsible for lipolysis, glycogenolysis, and proteolysis [41]. These levels decline during the day and activate catabolism and energy consumption. This might be one of the main reasons for the high glycolysis and oxidation during the awake phase [42]. This process supports ATP enough and the energetic demands associated with higher contractility. However, the increased protein turnover during the sleep period prevents damaged proteins in organelles (e.g., mitochondria) and cardiac function. Thus, during the REM phase, there is an intermediate glucose oxidation which is declined in the non-REM phase and upregulated in the awake [43]. While growth hormone levels are elevated in the slow sleep wave, cortisol is increased when waking up in the morning [44]. In fact, this situation provokes a higher blood pressure and help to some cardiac episodes such as infarction myocardium, ischemic events, or arrhythmias [45].

### 1.5. Hormonal Regulation

#### 1.5.1. Insulin

During the postprandial period, there is an increase in glucose, and in consequence, insulin is secreted by the pancreas. After insulin binding to its receptor, the kinase signaling (PKB/Akt) is activated and glucose transporter is translocated from cytosol into membrane. Then, glucose goes inside of cells [46]. Insulin also inhibits the adipose tissue triglyceride lipolysis with a consequent reduction of long-chain fatty acids release [47]. Although the most important role in cardiomyocytes is associated with glucose uptake, insulin can also upregulate the translocation into the surface membrane of the fatty acid translocase (FAT/CD36), the transporter for long-chain fatty acids, and the flow of fatty acids into the cytosol [48]. However, insulin deficiency is associated with an increment of fatty acid availability as an energy substrate in the heart where they will be oxidized [49]. Cardiac and skeletal muscle can uptake the free fatty acids from circulation because there is an increment of lipoprotein lipase synthesis on their cells, which is necessary for breaking down triglycerides. However, the decline of this lipoprotein lipase activity in adipose tissue [50] reduces the fatty acids uptake by this tissue. A longer deficiency of insulin activity on the cells can be caused by a reduction of its production or low insulin receptor substrate-1 (IRS-1) levels. The disequilibrium between serine/threonine and tyrosine phosphorylation of this receptor causes its degradation and consequently a reduction of insulin activity [51]. There are several factors which can contribute to this insulin receptor dysfunction, inflammation caused by pro-inflammatory cytokines, dietary fatty acids, or adipose tissue expansion [52,53]. Other important responses of insulin are related to vasodilatation, cell growth, or protein translation in cardiomyocytes. However, in contrast with other cells, insulin does not seem regulate the glycogen synthesis in cardiac cells, which is mediated by glucose-6-phosphate [54].

#### 1.5.2. Thyroid Hormones

Thyroid glands produce thyroid hormones (T3 and T4) through iodine and triiodothyronine and thyroxine, respectively. They have an important role in cardiac regulation because can modulate the transcription of some genes or because their metabolites can act as a messenger with their own effects. Some of them are diastolic and systolic functions, mediated by the iodothyronines, or cardioprotective functions, mediated by the thyronamines. Both are metabolites of thyroid hormones (TH) [55]. They can link with nuclear receptors of cardiomyocytes and can regulate cardiac gene expression. Moreover, the electrochemical and mechanical response of myocardium may be conditioned by plasma-membrane ion transporters. Also, they might increase the sensitivity of the sympathetic system or might modify cardiac contraction considering hemodynamic alterations [56]. Thyroid receptor dysfunction may limit the heart’s ability to shift substrate pathways and energy supply. For instance, 3-Iodothyronamine (T1AM), a metabolite of TH, was a non-competitive inhibitor of ATP synthase, in such a way, the electron chain does not produce ATP [57]. Hyperthyroidism attenuates the insulin-dependent glucose uptake, glycolysis, and glucose oxidation. However, hypothyroidism downregulates the free fatty acids oxidation because, while carnitine palmitoyl-transferase is decreased [58], there is an increment of pyruvate dehydrogenase kinase-2 [59,60]. The main consequence is the reduction of heart rate and diastolic function [61]. 

#### 1.5.3. Insulin Growth Factor 1 (IGF-1)

This hormone shares an 80% homology with insulin [62]. IGF-1 is a very small peptide mainly produced by the liver. Both proteins have the same downstream activity (PI3K/Akt) [63]. Akt has two isoforms: Akt1 is associated with growth, and Akt2 is associated with metabolism. In fact, growth hormone (GH) and insulin act with IGF-1 to induce the metabolic pathway [64,65] through glucose uptake. The GH/IGF-1 axis enhances the calcium intracellular and cardiomyocytes contractility. In obesity and type 2 diabetes mellitus (T2DM), IGF-1 can induce cardiomyocytes hypertrophy through overactivity of growth pathway [66].

#### 1.5.4. Growth Hormone

The growth hormone (GH) is secreted by somatotropic cells of the anterior pituitary gland, and insulin-like growth factor I (IGF-I) has an important role in heart development. The excess or deficiency of these hormones can be the cause of heart malformation. Nevertheless, these can be a potential treatment for some cardiac diseases [67]. Several studies highlight the importance of the balance GH/IGF-I [68]. GH stimulates the production of IGF-1. However, its receptor is expressed in cardiomyocytes and can decrease the glucose uptake and increment the fatty acids uptake and protein rate [69].

#### 1.5.5. Estrogen

Estrogens, which are steroid hormones and are produced by ovaries and adrenal glands, participate in carbohydrates and lipids metabolism [70]. They can bind to three known estrogen receptors (ER): ERα, ERβ, and G-protein-coupled estrogen receptor (GPR30 or GPER). The last one is only expressed in adult cardiomyocytes, mainly in ventricles [71], and can induce mitochondrial biogenesis after E2 binding [72]. The acute activation of ER can also induce the signal transduction of PI3K/Akt [73]. However, E2 also prevents insulin resistance and obesity because it reduces the lipogenic activity in adipose tissue [74]. 

#### 1.5.6. Cortisol

Cortisol is a glucocorticoid hormone which is released by the adrenal gland. Its production is increased in stressful conditions, and their overproduction causes changes in lipid metabolism and, in consequence, obesity and insulin resistance. There are enzymes, e.g., 11β-hydroxysteroid dehydrogenase (11β-HSD), involved on the conversion of inactive cortisone towards active cortisol. The isoform 1, 11β-HSD type 1 (11β-HSD1) acts in liver, adipose tissue, gonadal tissue, and the central nervous system. However, 11β-HSD2 is expressed in a number of tissues [75]. Then, a chronic stressful situation in our life may produce true diseases [76] and, during pregnancy, can produce metabolic alteration in newborn heart [77]. Cortisol binds to glucocorticoids receptors, which are expressed in adipose tissue [78]. While this hormone reduces glucose uptake and its metabolism [79], synergically, insulin improves the energy storage and lipogenesis [80] through acetyl-CoA-carboxylase and fatty acid synthase activation [81].

#### 1.5.7. GLP-1

The glucagon-like peptide-1 (GLP-1), produced by the gut in response to nutrients in the intestinal lumen increases the insulin synthesis and secretion by pancreas in a glucose-dependent manner and hepatic gluconeogenesis [82]. Thus, this hormone improves glucose sensitivity [83]. Its receptors were described in the heart [84], and through them, this peptide exerts the activation of cAMP/protein kinase A [85]. Some authors described that this hormone is able to increase the mitochondrial biogenesis through peroxisome proliferator-activated receptor gamma coactivator 1-alpha (PGCα) in heart cells [86] and through thermogenesis in brown adipocytes [87]. Preadipocytes also express GLP-1 receptors, and their levels are decreasing with the adipocyte’s differentiation. GLP-1 improves glucose uptake [88] and might upregulate oxidative activity through mitochondrial biogenesis. 

### 1.6. Ageing

Adult hearts need a high mitochondria number due to the oxidative phosphorylation activity [89]. During ageing, fatty acids transporters in the sarcolemma are enhanced, but the inability to oxidize them [90] causes lipid accumulation and lipotoxicity, such as palmitic acid, acylcarnitine, unesterified cholesterol, lysolecithin, ceramide, and diacylglycerides [91]. These lipids can trigger apoptosis and inflammation and can emphasize mitochondrial dysfunction. Thus, ageing is associated with a reduction of fatty acid oxidation [92,93] and an upregulation of glucose oxidation through pyruvate dehydrogenase [94]. Although some authors have also described a change in mitochondria number and complex activity of the electron transport chain. The mitochondria lose capacity to metabolize fatty acids or glucose, and the oxygen left produces reactive oxygen species [95]. They might trigger apoptotic signaling on cardiomyocytes and myocardium remodeling and might modify the intercell communication and metabolism [96]. The deficiency of oxidative metabolism in myocardium and the reduction of catecholamines, fasting, or exercise-induced lipolytic response [97,98] on adipose tissue contributes to the increment of epicardial fat [99]. However, there is also a high circulating free fatty acid due to the mitigation of insulin response and the decline of energy expenditure [100]. This situation could increase the glucose production and could impair insulin-dependent glucose uptake [101,102]. The increment of free fatty acids in the blood will contribute to the triglycerides-rich very low density lipoprotein (VLDL) particles in the liver [103] and cardiovascular disorders.

## 2. The Metabolism of Cardiomyocytes in Pathological Conditions

### 2.1. “Obesity” Cardiomyocytes and Metabolism

Obesity can produce changes in hemodynamic that cause heart morphology and metabolism alterations [104]. There is fatty acid accumulation and inflammation on adipose tissue [105,106]. They are the main causes of insulin resistance [107]. There is an increase of myocardial fatty acid uptake and a decline on myocardial efficiency (cardiac work/oxygen usage) [108]. The high fatty acid utilization produces mitochondrial uncoupling and energy wastage [109], inducing diastolic dysfunction [110]. The shift from fatty acids towards glucose substrate for getting energy is reduced because of the presence of insulin resistance [111]. The high fatty acids in the myocardium promote their accumulation of lipids and synthesis of triacylglycerol, diacylglycerol, and ceramide, which can also induce cardiomyocytes dysfunction; insulin resistance; and finally, apoptotic cells and death [112]. All these metabolic changes, excess of fatty acids used as energy fuel, starts a stress situation and chronic inflammation on adipose tissue. Although some authors have described the obesity paradox in heart failure [113], we should consider the metabolic disorder, nutritional stage, and fragility associated with heart failure progression in those lean patients with fatal events.

### 2.2. “Diabetes” Cardiomyocytes and Metabolism

There are different types of diabetes mellitus (DM) depending on the insulin production by the pancreas or the insulin effects on cells. The failure of the first option is named type 1 DM and the second is type 2 (T2DM). As it was described before, insulin is a released hormone as a consequence of high blood glucose levels. Insulin binds to the specific receptor in cells and activates the PKB/Akt signal pathway. [46]. DM can produce changes in cardiomyocyte metabolism. Because insulin resistance reduces glucose uptake, the cardiomyocytes have to use fatty acids as the main support of energy [114]. The following mechanisms are similar between obesity and insulin resistance. There is an accumulation of lipids through the transcription activity of the nuclear receptor superfamily, peroxisome proliferator-activated receptors (PPARs) [115,116]. 

The remaining insulin stimulates the sterol regulatory element-binding protein (SREBP)-1c in the liver or heart and converts glucose towards fatty acids and triglycerides. This mechanism develops the fatty liver disease [117] or lipid deposition in cardiomyocytes [118]. T2DM is intimately related to the amount of epicardial adipose tissue [119]. Insulin resistance in adipose tissue is correlated with the inflammation and its remodeling. There is a pro-inflammatory profile that triggers the M1 macrophages presence, and the glucose oxidative phosphorylation is replaced by glycolysis. This step provides lactate and pyruvate. This last product can be converted into acetyl-CoA, which can be used for lipogenesis [120] and increases the epicardial adipose tissue. 

### 2.3. Ischemic Cardiomyocytes and Metabolism

The main important change in ischemia is oxygen deficiency, the reduction of oxidative phosphorylation, and ATP reduction. Thus, there is an increment of glucose uptake and glycogen breakdown [121]. Glucose into the cell is converted into lactate through lactate dehydrogenase (LDH) activity during the glycolysis process. This conversion is also associated with a rise of H+ and, consequently intracellular acidosis, lactate accumulation, and less contractile work [122]. The reduction of intracellular pH entails a higher ATP amount for the sarcoplasmic Ca2+ pump [122]. Thus, after myocardial infarction in rat, there is a high glucose-6-phosphate dehydrogenase activity [123]. If the ischemia is moderate, fatty acid oxidation rate is normal. Even, the high catecholaminergic activity increases fatty acids during myocardial infarction, and they are used as an energy substrate with the residual oxygen [124]. The inhibition of fatty acids oxidation and the upregulation of glucose oxidation will decline the lactate production and the intracellular acidosis with myocardial benefits. Thus, some therapeutic drugs, trimetazidine and ranolazine, which reduced the fatty oxidation through mitochondrial long-chain 3-ketoacyl coenzyme A thiolase inhibition [125] have demonstrated benefits on exercise tolerance and improvements on ventricular function [126,127]. 

### 2.4. Hypertrophic Cardiomyocytes and Metabolism

The failing heart loses the capacity of ATP generation due to mitochondrial dysfunction. More ATP is needed as there are higher hypertrophic walls. Thus, this condition tries to shift the fatty acid into glucose oxidation because it gets faster and more efficient energy. However, the pathological situation with mitochondrial dysfunction and less oxygen consumption starts to produce reactive oxidative species which are involved in an inflammatory process and metabolic disorder progression. In fact, glucose through glycolysis will be converted into lactate [128]. Moreover, the protective response against hypertrophic process upregulates polyol pathway. However, its progression produces the reduction of aldose reductase and hypertrophic growth [129]. The other pathway for preventing cellular stress is the pentose phosphate pathway. There is an increment of glucose-6-P-dehydrogenase that lead the pentose phosphate pathway and reactive oxygen species accumulation [130].

**Figure 1:**
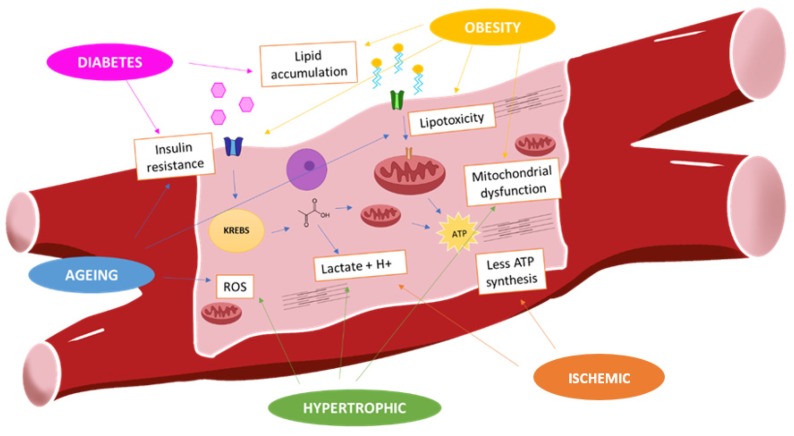
Myocardium metabolism in pathological situations: In physiological conditions, fatty acids are mainly fuel of energy of cardiomyocytes. Obesity is associated with high fatty acids uptake that develops lipotoxicity and insulin resistance. It reduces the glucose uptake. However, during hypertrophic and ischemic situation, there is shift from fatty acids to glucose that is converted into lactate. This metabolism gets less ATP production, and there is mitochondrial dysfunction and ROS (reactive oxygen species) production.

Thus, the cardiomyocytes get preferably energy from the available substrates, oxygen, or their transporters in pathological conditions. Table 1 and Figure 1 show the energy substrates in pathological conditions.

## 3. Epicardial Adipose Tissue Metabolism

**Figure 2:**
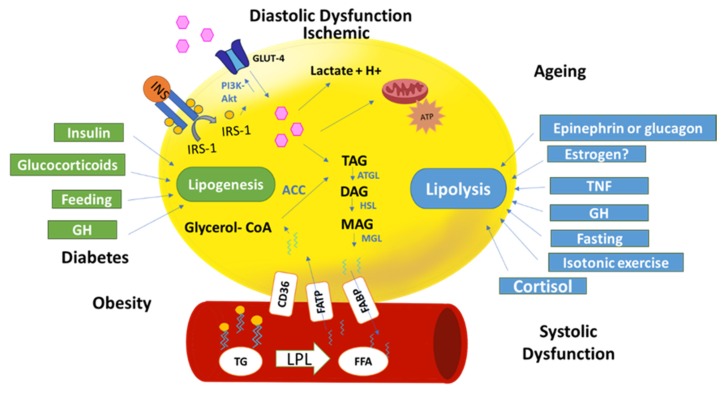
Epicardial fat metabolism in physiological and pathological situations: The figure represents an adipocyte (yellow) and a blood vessel. Glucose is the main fuel of energy in adipocytes to be used in adipocyte metabolism. Glucose or fatty acids can be stored in triacylglycerides (TAG) by a lipogenesis process. Insulin, glucocorticoids, and growth hormone (GH) promote lipogenesis. TAG through lipolysis produce free fatty acids (FFA). Several factors contribute to lipolysis (fasting, cortisol, etc.). Pathological conditions, diabetes, and obesity promote lipogenesis because of free fatty acid (FFA) uptake from the blood through fatty acids transporter protein (FATP). However, systolic dysfunction due, in part, by the epinephrine increment, promotes lipolysis. Diastolic and ischemic situation will increase glucose uptake for producing lactate and H+ due to low oxygen levels.

The main energetic metabolite of adipose tissue is glucose. Around 40% of glucose can be oxidized and used in de novo lipogenesis or glycogen synthesis. Twenty %–25% is used for synthesis of glycerol 3-phosphate and reesterified, and around of 30% is released as lactate [131,132]. Epicardial fat can develop a protective mechanism for storing the energy which is not used by the myocardium. The excess energy substrate can be associated with the myocardial dysfunction or high energy substrates intake. However, the high fat accumulation intensifies the proinflammatory activity on this tissue with high production of tumor necrosis factor (TNF-α), interleukin 1β, or IL-6 [133]. Thus, the epicardial fat thickness in the right atria-ventricle groove is an independent predictor for inflammatory state in metabolic syndrome [134]. TNF- α induces insulin resistance in terms of glucose uptake in myocytes and adipocytes. This cytokine upregulates the protein-tyrosine phosphatase expression levels which is involved in the dephosphorylation of phosphotyrosine residues of the insulin receptor and IRS-1 [135]. Because insulin is an anabolic hormone, since its signal transduction inhibits lipases and stimulates the fatty acids storage, the adipose tissue starts to release fatty acids towards the circulation, which can be uptaken by the myocardium for getting energy. However, the increment of circulating fatty acids provokes a cardiac metabolic dysfunction with insulin resistance consequences [136]. In this sense, the epicardial fat thickness is associated with insulin resistance in obese [137] and impaired fasting glucose [138] in nondiabetic subjects. Moreover, the epicardial adipose tissue can produce angiotensinogen and angiotensin II, which is able to interfere with the insulin signaling, to increase the oxidative stress, and to decrease the anti-inflammatory adipokine—adiponectin—levels [139]. It was demonstrated that the insulin resistance after cardiac surgery is related to the angiotensinogen [140], pro-inflammatory cytokines, and fibroblast growth factor 21 production [141] by epicardial fat [142]. Other findings regarding the expression of low-density lipoprotein receptor in epicardial fat with diabetes [143] suggest the association between epicardial fat and lipid metabolism disorder. Thus, the inflammatory state, the insulin resistance, and the lipid metabolism dysfunction might contribute to the reduction of differentiation ability of epicardial mesenchymal cells [144] and the production of anti-inflammatory adipokine, adiponectin [145]. Obesity and insulin resistance progression will contribute to coronary artery disease. Thus, the epicardial fat from these patients expresses low levels of glucose transporter, GLUT-4, and high levels of retinol-binding protein 4 (RBP4), associated with insulin resistance [146]. The released factors by this fat tissue can contribute to the impairment of insulin signaling in cardiomyocytes and its contractility [147]. The adipose tissue accumulation can enhance the production of released proteins-induced myocardial fibrosis [148] and the reduction of distensibility and ventricular dysfunction. In addition, heart failure is associated with an increment of catecholamines and natriuretic peptides [149]. Catecholamines have a catabolic and lipolytic signal in adipose tissue through β3-adrenergic receptors. Then, there is an activation of adenylate cyclase and increase in adenosine monophosphate synthesis which is involved in protein kinases activation. This downstream signal transduction activates lipases and triglycerides breakdown into fatty acids and glycerol [150]. The natriuretic peptides can also activate lipases from the guanylyl cyclase-A receptor, which can activate guanylate cyclase and cyclic GMP production. This molecule allows the activation of protein kinase G and lipases [151]. Thus, some studies have demonstrated the inverse association between epicardial fat volume and left ventricular systolic dysfunction in patients with heart failure [152]. Some explanations of this phenomenon might be focused on fatty acid mobilization, since a high expression levels of the fatty transporter, fatty acid binding protein 3 (FABP3), was detected in epicardial fat from patients with heart failure [153], or on dysfunction for glucose uptake [154]. These processes might explain the lower amount of epicardial fat in patients with congestive heart failure [155]. A brief summary is represented in Figure 2.

## 4. Hormonal Therapy on Cardiac Metabolism

### 4.1. Estrogen Therapy

In spite of adipose tissue, during the menopausal stage, estrogens produced from androgens conversion through aromatase activity [156] do not reach the physiological estrogen levels. Thus, the deficiency tried to be replaced by hormone therapy in menopausal women. The KEEPS (Kronos Early Estrogen Prevention Study) trial has demonstrated the oral estrogen therapy effects on epicardial fat reduction without cardiovascular risk [157]. This therapy improves the glucose metabolism and declines fatty acids uptake through lipoprotein lipase downregulation by estrogen response element at the promoter region of the gene [158]. Additionally, estrogens upregulate hormone-sensitive lipase expression in a catecholamines-dependent manner and free fatty acids are released into circulation [159]. However, lipolysis does not occur in subcutaneous fat [160], where there is an upregulation of the antilipolytic receptor, α2A adrenergic [161]. In this sense, estradiol treatment might cause subcutaneous fat accumulation and the mitigation of epicardial fat with benefits on cardiovascular disease. However, the estrogens with progestin [162] or raloxifene, an estrogen receptor modulator [163], therapy did not show coronary heart disease benefits and risk of thromboembolism events. The differential behavior of estrogen receptors with dependence on adipocytes differentiation and localization might explain the controversial effects of the clinical trials. 

### 4.2. GLP-1 Receptor Agonists

The cardiovascular benefits of GLP-1 therapy were described in several cohorts of patients [164], those undergoing open heart surgery [164] or percutaneous coronary intervention [165] or those diagnosed with T2DM [166]. Some of the mechanism involved, in this protective effect of GLP-1, were associated with the reduction of inflammation, CD36 translocation, fatty acids uptake, and improvement of glucose uptake through Akt activity [167,168]. 

Some authors have also studied the effect of this therapy on epicardial adipose tissue volume since GLP1 receptors (type 1 and type 2) were used to described it [169], and they are associated with fatty oxidation-related genes [170,171]. Most of the observational studies demonstrated the reduction of epicardial fat after GLP-1 therapy [172,173]. These results might be discussed since some trials did not detect the ectopic fat reduction after GLP-1 therapy [174]. However, there is a decrease in oxidative stress and endothelial inflammation [175], which might be increased by epicardial adipose tissue accumulation 

## 5. Solutions and Nutritional Therapies on Metabolic Signaling

### 5.1. Polarizing Solutions

A polarizing solution or glucose-insulin-potassium (GIK) is a therapy for infarction; this produces changes in cardiomyocytes metabolism and guides the cells to use more glucose than lipids [176]. This one obtains a cardioprotective effect on the ischemic event because it promotes the glucose uptake through GLUT-1 and GLUT-4 [177]. The solution improves oxidative phosphorylation, restores the energy, and enhances the aerobic and anaerobic glucose metabolism. Potassium improves the insulin action and glucose uptake and reestablishes the intracellular ions and contractility of cardiomyocytes. This solution was used on open-heart surgeries [178,179]. Another important effect is the decrease of circulating free fatty acids [180], which can be explained by the lipogenic effect of insulin. 

### 5.2. Coenzyme Q10

Coenzyme Q10 [181] exists in reduced or antioxidant form. This molecule is localized in the mitochondrial inner membrane and acts in the electron transport chain for ATP production. Thus, it increases the production of ATP and reduces the oxidative stress, the membrane oxidation, and lipid peroxidation. Moreover, it stabilizes the calcium-dependent ion channel in the myocardium. Its supplementation in patients with cardiopathies, caused by the coenzyme Q10 deficit, showed a cardioprotective effect [182] or reduced mortality in elderly subjects [183]. However, the designed clinical trials with preserved heart failure patients failed to show the benefits of coenzyme Q supplementation [184] in left ventricle dysfunction.

### 5.3. L-carnitine

Endogenous L-Carnitine is an enzyme which is located in mitochondrial membrane and which translocates the long-chain fatty acids into the mitochondrial matrix for their oxidation. Some human tissues, cardiac or muscle, have more levels of carnitine than they need for fatty acids oxidation [185]. L-carnitine can also be obtained by diet, in meat or fish. This one has a different function than endogenous carnitine. Mainly, it reduces oxidative stress of cells and could be useful for some cardiovascular diseases [186]. Carnitine deficiency produces lipid metabolism diseases and other disorders associated with membrane permeability or lipid peroxidation. They might exacerbate myocardial diseases. For this reason, the supplementation of L-carnitine was suggested to be cardiovascular protective [187]. However, the American guidelines did not recommend its use in patients with heart failure with or without reduced ejection fraction [188].

## 6. Others Metabolic Therapies

### 6.1. Trimetazidine

This drug reduced fatty acid oxidation by blocking the activity of 3-ketoacyl-coenzyme A thiolase, which inhibits the pyruvate dehydrogenase. The main consequence is the shift from fatty acids metabolism into glucose utilization [189] and preserves the intracellular phosphocreatine and ATP ratio. This situation, as it was described before, is a protective mechanism against ischemia myocardial injury [190,191]. There is a reduction of resting energy expenditure, which is associated with the increment of catecholamines and growth hormone and the decrease of left ventricle function [192], all factors associated with heart failure. Thus, patients with chronic heart failure and treated with trimetazidine showed an 11% mortality reduction [193].

### 6.2. SGLT2 Inhibitors

SGLT2 is a sodium-glucose cotransporter, which is mainly expressed in kidneys. Its inhibition increases sodium and glucose excretion. Then, the main action of the inhibitor of SGLT2 (SGLT2i) is the improvement of glycaemic status. The results of the clinical trials have shown cardiovascular benefits [194,195,196]. If SGLT2i modulates the glucose levels, their protective role might involve a metabolic change in myocardium. However, there are controversial studies regarding metabolic substrate preference by myocardium after SGLT2 inhibitors treatment [197]. The main difference in these studies is the animal models use for testing their effects (specie, fast state, disease, etc.). All of them are very important factors associated with the metabolic substrate preference. However, the highlight is that this drug improves the metabolic efficiency [198,199], which is necessary for cardiomyocytes contractility under low oxygen conditions. 

### 6.3. Metformin

This antidiabetic protein phosphorylates the key regulatory site (Thr-172) on the catalytic (α) subunit of AMPK in cardiomyocytes [200]. This protein regulates the energetic homeostasis and inhibits the anabolic pathways. This molecule promotes lipoprotein lipase, translocates fatty acid transporters into sarcolemma, and increases fatty acids uptake and oxidation [201]. AMPK is also activated in stressful conditions and glucose deprivation [202] for increase in glucose metabolism and mitochondrial biogenesis. An observational trial in diabetic patients under metformin treatment reduced the epicardial adipose tissue thickness [203]. These process in combination with the anti-inflammatory and antioxidant effects might explain the protective benefits on diabetic with cardiovascular disease [204].

## 7. Conclusions

The myocardium and epicardial adipose tissue have an important role to get and store energy, respectively. The metabolic dysfunction of myocardium reflects high epicardial adipose tissue volume, which is associated with an inflammatory state, glucose resistance, and free fatty acid release. The improvement on glucose oxidative metabolism will reduce epicardial fat, and myocardium will get energy in a more efficient way in pathological conditions (low oxygen supply and low mitochondrial number). 

## Figures and Tables

**Table 1:** Energy substrates preferences of myocardium on pathological conditions.

Pathological Conditions	Energy Substrate
Obesity	Fatty acids
Diabetes	Fatty acids
Ischemic cardiomyopathy	Glucose, lactate
Hypertrophic cardiomyopathy	Glucose

## Abbreviations

ATP	adenosine triphosphate
ATPase	adenosine triphosphatase
NCX	sarcolemmal Na^+^/Ca^2+^ exchanger
FFA	Free fatty acids
FADH	Flavin adenine dinucleotide
NADH	Nicotinamide adenine dinucleotide (NAD) + hydrogen (H)
GLUT	Glucose transporter
LDH	lactate dehydrogenase 4
BMAL1	Brain and muscle Arnt-like protein-1
AMPK	adenosine monophosphate-activated protein kinase
IGF-1	insulin growth factor
GLP1	glucagon like peptide 1
SGLT2	sodium glucose transporter 2
